# O Prolongamento do Intervalo QTc na Admissão está Associado ao Aumento da Mortalidade em Pacientes com SARS-COV-2 durante a Hospitalização

**DOI:** 10.36660/abc.20220155

**Published:** 2022-12-20

**Authors:** Stephany Barbosa, Oscar Mauricio Muñoz, Alejandra Cañas, Angel Alberto Garcia

**Affiliations:** 1 Departamento de Medicina Interna Pontificia Universidad Javeriana Hospital Universitario San Ignacio Bogotá Colômbia Departamento de Medicina Interna - Pontificia Universidad Javeriana – Hospital Universitario San Ignacio, Bogotá – Colômbia

**Keywords:** COVID-19, Mortalidade, Eletrocardiografia

## Abstract

**Fundamento:**

O envolvimento cardiovascular associado à infecção por SARS-COV-2 está relacionado a desfechos desfavoráveis durante a internação. Portanto, a medida na admissão do intervalo QTc no eletrocardiograma de 12 derivações pode ser um marcador prognóstico.

**Objetivo:**

Identificar a relação entre o prolongamento do QTc na admissão durante a hospitalização e a mortalidade por SARS-COV-2.

**Método:**

Estudo observacional baseado em uma coorte retrospectiva de pacientes com infecção confirmada por SARS-COV-2 do Hospital Universitário San Ignacio, Bogotá (Colômbia), entre 19 de março de 2020 e 31 de julho de 2021. A mortalidade foi comparada em pacientes com QTc prolongado e normal na admissão e controle das variáveis clínicas e comorbidades por meio de modelos de regressão logística bivariada e multivariada. Um valor de p <0,05 foi considerado estatisticamente significativo

**Resultados:**

Foram analisados 1.296 pacientes e 127 (9,8%) apresentaram QTc prolongado. A mortalidade foi maior em pacientes com QTc prolongado (39,4% vs. 25,3%, p=0,001), assim como o tempo de internação (mediana 11 vs. 8 dias; p=0,002). Na análise multivariada, a mortalidade foi associada a QTc prolongado (OR 1,61, IC 95%: 1,02; 2,54, p=0,038), idade (OR 1,03, IC 95% 1,02; 1,05, p<0,001), sexo masculino (OR 2,15, IC 95% 1,60; 2,90, p<0,001), doença renal (OR 1,32, IC 95% 1,05; 1,66, p=0,018) e índice de comorbidade de Charlson > 3 (OR 1,49, IC 95% 1,03; 2,17, p=0,035).

**Conclusões:**

A mortalidade hospitalar por SARS-COV-2 está associada ao prolongamento do intervalo QTc no momento da admissão, mesmo após ajuste para idade, sexo, comorbidades e gravidade basal da infecção. Pesquisas adicionais são necessárias para estabelecer se esses achados estão relacionados ao envolvimento cardíaco pelo vírus, hipóxia e inflamação sistêmica.

## Introdução

A mortalidade em pacientes com infecção por SARS-COV-2 em todo o mundo é próxima de 2%.^1^ O número de casos continua a aumentar, com mais de 250 milhões de casos relatados até novembro de 2021.^2^ O envolvimento cardiovascular associado à infecção por SARS-COV-2 está associado a pior prognóstico^3^ e aumento da mortalidade. Estima-se que aqueles com doença cardiovascular tenham um risco 3 vezes maior de sofrer da doença na forma grave.^4^ Em particular, é descrita uma alta prevalência de arritmias cardíacas em pacientes infectados, sugerindo haver envolvimento específico do miocárdio ou do sistema de condução pelo vírus,^5^ mediado principalmente por processos inflamatórios e hipóxia.^6^

O eletrocardiograma é uma ferramenta útil para avaliar o grau de acometimento cardíaco por sua ampla disponibilidade, baixo custo, natureza não invasiva e possibilidade de seguimento imediato.^7^ Tem sido descrito que até 90% dos pacientes hospitalizados^8^ apresentam alterações eletrocardiográficas, incluindo taquicardia sinusal, prolongamento do intervalo QT, Torsades de Pointes, fibrilação atrial, alterações inespecíficas do ST e a onda T, bem como alterações de condução ou arritmias ventriculares.

O prolongamento do intervalo QT corrigido (QTc) depende da variação da despolarização e da repolarização ventricular.^9^ No entanto, se desconhece a relevância clínica desses achados eletrocardiográficos associados à infecção por SARS-COV-2, já que além de estarem relacionados a arritmias, podem se tornar um marcador de risco útil.^10 , 11^

Este estudo visa identificar a relação entre prolongamento do QTc na hospitalização e a mortalidade por SARS-COV-2 durante a hospitalização, controlado por fatores como idade, sexo e comorbidades, com base em uma coorte de pacientes atendidos em um hospital de alta complexidade em Bogotá, Colômbia.

## Método

Um estudo observacional analítico foi realizado em uma coorte retrospectiva baseada no registro institucional de pacientes com SARS-COV-2 atendidos no Hospital Universitário San Ignacio (HUSI) em Bogotá, Colômbia, entre 19 de março de 2020 e 31 de julho de 2021. Foram incluídos todos os pacientes maiores de 18 anos internados por infecção por SARS-COV-2 confirmada por teste de PCR, que tiveram eletrocardiograma de 12 derivações realizado na entrada da internação. O Comitê de Ética e Pesquisa da Pontifícia Universidade Javeriana e do Hospital Universitário San Ignacio aprovaram o estudo (Código de Aprovação: FM-CIE-0883-21).

As informações sobre variáveis sociodemográficas, comorbidades, laboratórios de admissão e desfechos foram sistematicamente coletadas e armazenadas no registro institucional confidencial por meio do RedCap®. A taxa de perda de dados foi monitorada para garantir a qualidade dos mesmos e as informações dos dados extremos foram verificadas.

### Análise estatística

Para a análise, a população foi dividida em dois grupos: pacientes com intervalo QTc dentro dos limites da normalidade (QTc homens < 470 mseg e QTc mulheres < 480 mseg) e o segundo grupo com pacientes com intervalo QTc prolongado.

O teste de Shapiro-Wilk foi utilizado para avaliar a suposição de normalidade. As variáveis contínuas não apresentaram distribuição normal; portanto, descrevemos a mediana e o intervalo interquartil (IIQ). A comparação entre as características dos grupos foi realizada por meio do teste Qui-quadrado para variáveis categóricas ou teste U de Mann-Whitney para variáveis contínuas. Um valor de p menor que 0,05 foi considerado estatisticamente significativo.

Foi realizada uma análise de regressão logística, tendo como desfecho a mortalidade. Inicialmente, foi realizada uma análise bivariada e, posteriormente, uma análise multivariada. O impacto do prolongamento do QTc basal foi avaliado controlando as variáveis associadas à mortalidade descritas em estudos anteriores, como idade, sexo; comorbidades como hipertensão arterial, infarto do miocárdio, insuficiência cardíaca, diabetes mellitus, doença renal crônica (DRC), doença pulmonar obstrutiva crônica (DPOC), índice de Charlson (ferramenta de avaliação projetada especificamente para prever mortalidade a longo prazo que tem sido usada para demarcar diferenças prognósticas entre subgrupos de pacientes que compartilham o mesmo diagnóstico médico)^12^ e uso de medicamentos que prolongam o intervalo QTc (hidroxicloroquina, cloroquina, azitromicina, lopinavir/ritonavir). Um valor de p menor que 0. 05 foi considerado estatisticamente significativo. A análise foi realizada usando um pacote estatístico StataCorp. 2019. (Stata Statistical Software: Release 16. College Station, TX: StataCorp LLC).

## Resultados

Foram incluídos 1.296 pacientes com indicação de internação por SARS-COV-2, dos quais 127 (9,8%) apresentavam intervalo QTc prolongado no eletrocardiograma realizado na admissão. A Tabela 1 resume as características demográficas e clínicas no momento da admissão, comparando os grupos de acordo com o intervalo QTc basal. As idades não apresentaram diferenças estatisticamente significativas. O sexo masculino foi mais frequente entre os pacientes com intervalo QTc prolongado. A permanência hospitalar foi maior no mesmo grupo, com mediana de 11 (IIQ 6 - 19) vs. 8 dias (IIQ 4 - 16). A mortalidade durante a internação foi menor em pacientes com QTc normal (25,3% vs. 39,4%). Um ecocardiograma foi realizado durante a internação em 241 pacientes (18,6%). Destes, a fração de ejeção foi superior a 50% em 58,6% e <40% em 22,6%, com características semelhantes nos dois grupos.

**Tabela 1:** Características clínicas e demográficas dos pacientes incluídos de acordo com o QTc basal

Variável	QTc normal n = 1169	QTc prolongado n = 127	Valor-p
Idade, anos, mediana (IIQ)	63 (52-74)	67 (54-75)	0,065
Masculino, n (%)	637 (54,5)	81 (61,8)	0,045
**Comorbidades, n (%)**			
Pressão alta	480 (55,6)	67 (68,4)	0,016
Infarto do miocárdio	71 (8,2)	8 (8,2)	0,983
Insuficiência cardíaca	87 (10,1)	18 (18,4)	0,013
Diabetes Mellitus	208 (24,1)	24 (24,5)	0,932
Doença renal	89 (10,3)	15 (15,3)	0,132
DPOC	113 (13,1)	15 (15,3)	0,541
Charlson > 3, n (%)	362 (31,0)	41 (32,3)	0,761
**Laboratórios na admissão, Mediana (IIQ)**			
LDH, U/L	318 (237-448)	414(288-552)	<0,001
PCR, mg/dl	10,5(4,5-17,4)	10,5(7,3-18,7)	0,214
Dímero D, FEU/mL	871 (532-1553)	1233(659-2203)	0,006
Medicamentos*	73 (6,2)	5 (4,0)	0,299
Dias de internação, mediana (IIQ)	8 (4-16)	11 (6-19)	0,002
Óbito hospitalar, n (%)	296 (25,3)	50 (39,4)	0,001

DPOC: doença pulmonar obstrutiva crônica; IIQ: intervalo interquartil; U/L: unidades por litro; mg/dl: miligrama por decilitro; FEU/mL: unidades equivalentes de fibrina por mililitro. *Pelo menos 1 medicamento que prolonga o QTc. LDH: lactato deshidrogenase; PCR: proteína c-reactiva.

Em relação às comorbidades dos pacientes incluídos ( Tabela 1 ), a hipertensão arterial foi a mais frequente, seguida do diabetes mellitus. O índice de comorbidades de Charlson foi semelhante nos dois grupos. Entretanto, uma maior frequência de hipertensão arterial e insuficiência cardíaca foi encontrada no grupo de pacientes com intervalo QTc prolongado. Os valores medianos de LDH e D-dímero na admissão foram significativamente maiores entre os pacientes com prolongamento do QTc. Não houve diferenças significativas no uso de medicamentos que prolongaram o QTc.

A análise bivariada ( Tabela 2 ) mostrou aumento significativo da mortalidade associada ao prolongamento do intervalo QTc, idade e sexo masculino. As comorbidades associadas ao aumento da mortalidade foram: hipertensão, doença renal, DPOC e índice de Charlson > 3.

**Tabela 2:** Análise bivariada e multivariada de todos os pacientes incluídos

Variável	Análise bivariada		Análise multivariada	

	OR (IC 95%)	Valor-p	OR (IC 95%)	Valor-p
QTc prolongado*	1,91 (1,31–2,80)	0,001	1,61 (1,02-2,54)	0,038
Anos de idade	1,05 (1,04-1,06)	< 0,001	1,03 (1,02-1,05)	< 0,001
Masculino**	1,87 (1,45-2,43)	< 0,001	2,15 (1,60-2,90)	< 0,001
**Comorbidades**				
Pressão alta	1,47 (1,12-1,95)	0,006	0,90 (0,65-1,25)	0,560
Infarto do miocárdio	1,01 (0,62-1,66)	0,946	0,63 (0,37-1,09)	0,102
Insuficiência cardíaca	1,19 (0,78-1,83)	0,402	0,70 (0,43-1,14)	0,157
Diabetes Mellitus	1,30 (0,95-1,77)	0,094	1,23 (0,89-1,72)	0,215
Doença renal	1,45 (1,17-1,77)	< 0,001	1,32 (1,05-1,66)	0,018
DPOC	1,81 (1,24-2,64)	0,002	1,13 (0,74-1,72)	0,559
Charlson >3	1,33 (1,27-1,40)	< 0,001	1,49 (1,03-2,17)	0,035
Medicamentos***	1,59 (0,98-2,56)	0,060	1,04 (0,61-0,13)	0,871

DPOC: doença pulmonar obstrutiva crônica; OR: razão de possiblidades; IC: intervalo de confiança. * QTc > 480 milissegundos. ** Em comparação com as mulheres. *** Pelo menos 1 medicamento que prolonga o QTc.

Na análise multivariada ( Tabela 2 ), houve associação significativa entre mortalidade e prolongamento do intervalo QTc após controle para idade, sexo masculino, doença renal e índice de comorbidade de Charlson >3. As demais variáveis incluídas não apresentaram significância estatística na análise multivariada.

## Discussão

Nosso estudo é o primeiro realizado na América Latina e o maior relatado até o momento, no qual avaliamos a relação entre mortalidade intra-hospitalar e prolongamento do intervalo QTc na admissão. Encontramos uma diferença estatisticamente significativa na mortalidade em pacientes com QTc prolongado na admissão após o controle de idade, sexo e comorbidades.

Nossos resultados são semelhantes aos relatados por Farré et al., que analisaram 623 pacientes com SARS-COV-2, onde 9,8% apresentavam QT prolongado, e constataram que a morte por qualquer causa foi maior nesses pacientes (41,0% versus 8,7%, p < 0,001, HR 2,68, IC 1,58; 4,55).^10^ Além disso, eles apoiam os resultados reportado por Alsagaff de uma metanálise que incluiu sete estudos, para um total de 2.539 pacientes com infecção por SARS-COV-2, em que foi relatado um desfecho final composto por internação em UTI, doença grave e/ou mortalidade mais frequente entre pacientes com prolongamento do intervalo QTc, (DMP 6,04; IC 95%: 2,62: 9,45, p = 0,001; I^2^: 0%).^13^

Tem sido amplamente descrito que a inflamação sistêmica e a hipóxia na infecção por SARS-COV-2 podem induzir anormalidades na condução cardíaca.^14 , 15^ Foram até descritas como um marcador simples que reflete o estado inflamatório no nível celular miocárdico.^16^ Em nosso estudo, pacientes com prolongamento do intervalo QTc apresentaram valores basais adicionais de marcadores inflamatórios como LDH e marcadores de dano endotelial com envolvimento trombótico, como o D-dímero, o que reforça esta hipótese. Thakore et al. relataram que pacientes com intervalos QTc prolongados tiveram uma sobrevida menor durante a internação quando comparados a pacientes com QTc normal.^17^ Isso pode ser explicado por fenômenos imunomediados causados pelo vírus que resultam em uma tempestade de citocinas com níveis elevados de interleucina-6 (IL- 6),^18 , 19^ que bloqueia o canal de potássio éter-a-go-go, que por sua vez aumenta os níveis circulantes de IL-6, o acima foi amplamente reconhecido em doenças inflamatórias como a artrite reumatóide que estão associadas ao prolongamento do intervalo QTc e que melhoram com o manejo farmacológico, por exemplo, tocilizumabe (anticorpo monoclonal anti-IL-6).^20 , 21^

O uso de medicamentos que prolongam o intervalo QTc não apresentou diferenças estatisticamente significativas na mortalidade hospitalar, sugerindo que o prolongamento do QTc vai além de um efeito colateral de origem farmacológica.^16 , 22^ Jiménez-Jáimez et al. analisaram 219 pacientes com eletrocardiograma na admissão. Eles descrevem que pacientes ambulatoriais que não adoecem gravemente com SARS-COV-2 tratados com hidroxicloroquina, azitromicina e antirretrovirais desenvolvem um prolongamento não relevante do intervalo QT.^23^

Em nosso estudo, também encontramos uma relação entre o prolongamento do intervalo QTc e um aumento significativo nos dias de internação, o que pode estar relacionado à maior gravidade e complicações associadas. Outros autores sugeriram um achado, como Thakore et al., que descrevem maior duração do intervalo QTc em pacientes hospitalizados em comparação com pacientes que receberam alta (450,1 ± 30,2 vs. 423,4 ± 21,7 mseg, p < 0,0001).^17^

Encontramos na análise bivariada que a mortalidade hospitalar esteve associada a: idade avançada, sexo masculino, hipertensão arterial, doença renal crônica, DPOC e índice de Charlson maior que 3, achados que foram significativos na análise multivariada para idade, sexo masculino, doença renal crônica, e índice de Charlson maior que 3, semelhante ao relatado por Türkay et al., que incluíram 419 pacientes em seu estudo e os dividiram entre pacientes sobreviventes e não sobreviventes durante a internação por SARS-COV-2 e descreveram os achados paraclínicos identificados no pronto-socorro que poderiam prever a mortalidade, entre estes, pacientes com intervalos QTc mais longos, geralmente não sobreviviam.^24^

Nossos resultados sugerem que o aumento da mortalidade relacionada ao prolongamento do intervalo QTc foi independente da presença de comorbidades na admissão. Além disso, Al-Zakhari et al. analisaram retrospectivamente 339 pacientes e demonstraram que o intervalo QTc prolongado tem uma relação independente e estatisticamente significativa com a mortalidade em pacientes hospitalizados por SARS-COV-2 (r = -0,173, p<0,05, 95% IC: -0,277, -0,064).^25^

Nosso estudo contribui para o crescente conhecimento médico relacionado aos efeitos da infecção por SARS-COV-2 no sistema de condução cardíaco e à relação independente do QTc prolongado do eletrocardiograma de admissão como marcador prognóstico da doença, sendo um método econômico, disponível e fácil de interpretar na sala de emergência.

Como pontos fortes deste estudo, podemos citar um tamanho amostral significativo, derivado do maior banco de dados por SARS-COV-2 relatado até agora na Colômbia, com excelente qualidade e disponibilidade de informações, além da ausência de dados perdidos. As principais limitações descrevem vieses relacionados à fonte de informação, como o autorrelato de comorbidades e a possibilidade de subestimar a real prevalência de comorbidades nessa população. No entanto, se houver um viés de classificação incorreta, isso seria não diferencial, o que poderia diluir o tamanho do efeito em vez de aumentá-lo. Outra limitação é que o registro dos dados para avaliação da obesidade, comorbidade fortemente relacionada à mortalidade,^26^ foi insuficiente, por isso não foi incluído na análise. Da mesma forma, informações relacionadas ao prolongamento do intervalo QTc antes da infecção ou seu comportamento após a infecção não estão disponíveis. É uma limitação do nosso estudo que não pudemos identificar se o pior desfecho clínico associado ao QTc prolongado estava relacionado à disfunção ventricular, arritmias ventriculares ou dano estrutural cardíaco na linha de base, pois realizamos um ecocardiograma apenas em uma pequena fração dos pacientes. No entanto, nosso principal objetivo foi avaliar um fator associado à maior mortalidade, facilmente identificável no início da internação, ao invés de avaliar o mecanismo dessa associação. Estudos prospectivos adicionais seriam necessários para esclarecer esse ponto.

## Conclusões

A mortalidade hospitalar por SARS-COV-2 está associada ao prolongamento do intervalo QTc no momento da admissão, mesmo após ajuste para idade, sexo, comorbidades e gravidade basal da infecção. Pesquisas adicionais são necessárias para estabelecer se esses achados estão relacionados ao envolvimento cardíaco pelo vírus, hipóxia e inflamação sistêmica.

## References

[1] Díaz Pinzón JE (2021). Lethality by SARS-COV-2 worldwide. Rev Repert Med and Surgery.

[2] Ritchie H, Mathieu E, Rodés-Guirao L, Appel C, Giattino C, Ortiz-Ospina E (2021). Coronavirus Pandemic (COVID-19) – The Data.

[3] Madjid M, Safavi-Naeini P, Solomon SD, Vardeny O (2020). Potential Effects of Coronaviruses on the Cardiovascular System: A Review. JAMA Cardiol.

[4] Yang J, Zheng Y, Gou X, Pu K, Chen Z, Guo Q (2020). Prevalence of Comorbidities and its Effects in Patients Infected with SARS-CoV-2: A Systematic Review and Meta-analysis. Int J Infect Dis.

[5] Dherange P, Lang J, Qian P, Oberfeld B, Sauer WH, Koplan B (2020). Arrhythmias and COVID-19: A Review. JACC Clin Electrophysiol.

[6] Shi S, Qin M, Shen B, Cai Y, Liu T, Yang F (2020). Association of Cardiac Injury With Mortality in Hospitalized Patients With COVID-19 in Wuhan, China. JAMA Cardiol.

[7] Haseeb S, Gul EE, Çinier G, Bazoukis G, Alvarez-Garcia J, Garcia-Zamora S, Lee S (2020). Value of Electrocardiography in Coronavirus Disease 2019 (COVID-19). J Electrocardiol.

[8] Long B, Brady WJ, Bridwell RE, Ramzy M, Montrief T, Singh M (2021). Electrocardiographic Manifestations of COVID-19. Am J Emerg Med.

[9] Öztürk F, Karaduman M, Çoldur R, İncecik Ş, Güneş Y, Tuncer M (2020). Interpretation of Arrhythmogenic Effects of COVID-19 Disease Through ECG. Aging Male.

[10] Farré N, Mojón D, Llagostera M, Belarte-Tornero LC, Calvo-Fernández A, Vallés E (2020). Prolonged QT Interval in SARS-CoV-2 Infection: Prevalence and Prognosis. J Clin Med.

[11] Kochi AN, Tagliari AP, Forleo GB, Fassini GM, Tondo C (2020). Cardiac and Arrhythmic Complications in Patients with COVID-19. J Cardiovasc Electrophysiol.

[12] Charlson ME, Carrozzino D, Guidi J, Patierno C (2022). Charlson Comorbidity Index: A Critical Review of Clinimetric Properties. Psychother Psychosom.

[13] Alsagaff MY, Oktaviono YH, Dharmadjati BB, Lefi A, Al-Farabi MJ, Gandi P (2021). Electrocardiography on Admission is Associated with Poor Outcomes in Coronavirus Disease 2019 (COVID-19) patients: A systematic review and meta-analysis. J Arrhythm.

[14] Antwi-Amoabeng D, Beutler BD, Singh S, Taha M, Ghuman J, Hanfy A (2021). Association Between Electrocardiographic Features and Mortality in COVID-19 Patients. Ann Noninvasive Electrocardiol.

[15] Jain S, Workman V, Ganeshan R, Obasare ER, Burr A, DeBiasi RM (2020). Enhanced Electrocardiographic Monitoring of Patients with Coronavirus Disease 2019. Heart Rhythm.

[16] Merino JL, Martínez-Cossiani M, Iniesta A, Escobar C, Rey JR, Castrejón-Castrejón S (2020). COVID-19 and QT Interval Prolongation: More Than Just Drug Toxicity?. Europace.

[17] Thakore A, Nguyen J, Pollack S, Muehlbauer S, Chi B, Knight D (2021). Electrocardiographic Manifestations of COVID-19: Effect on Cardiac Activation and Repolarization. EClinicalMedicine.

[18] Lazzerini PE, Acampa M, Capecchi PL, Fineschi I, Selvi E, Moscadelli V (2015). Antiarrhythmic Potential of Anticytokine Therapy in Rheumatoid Arthritis: Tocilizumab Reduces Corrected QT Interval by Controlling Systemic Inflammation. Arthritis Care Res (Hoboken).

[19] Lazzerini PE, Boutjdir M, Capecchi PL (2020). COVID-19, Arrhythmic Risk, and Inflammation: Mind the Gap!. Circulation.

[20] Wu KC, Bhondoekhan F, Haberlen SA, Ashikaga H, Brown TT, Budoff MJ (2020). Associations Between QT Interval Subcomponents, HIV Serostatus, and Inflammation. Ann Noninvasive Electrocardiol.

[21] Lazzerini PE, Laghi-Pasini F, Acampa M, Boutjdir M, Capecchi PL (2020). IL-6 (Interleukin 6) Blockade and Heart Rate Corrected QT Interval Prolongation in COVID-19. Circ Arrhythm Electrophysiol.

[22] Wu CI, Postema PG, Arbelo E, Behr ER, Bezzina CR, Napolitano C (2020). SARS-CoV-2, COVID-19, and Inherited Arrhythmia Syndromes. Heart Rhythm.

[23] Jiménez-Jáimez J, Macías-Ruiz R, Bermúdez-Jiménez F, Rubini-Costa R, Ramírez-Taboada J, Flores PIG (2020). Absence of Relevant QT Interval Prolongation in Not Critically Ill COVID-19 Patients. Sci Rep.

[24] Kunt AT, Kozaci N, Torun E (2021). Mortality Predictors in Patients Diagnosed with COVID-19 in the Emergency Department: ECG, Laboratory and CT. Medicina (Kaunas).

[25] Al-Zakhari R, Atere M, Lim W, Abdulrahman M, Akhtar S, Sheets N (2021). Corrected QT Interval Prolongation, Elevated Troponin, and Mortality in Hospitalized COVID-19 Patients. Cardiol Res.

[26] Palaiodimos L, Kokkinidis DG, Li W, Karamanis D, Ognibene J, Arora S (2020). Severe Obesity, Increasing Age and Male Sex are Independently Associated with Worse In-hospital Outcomes, and Higher In-hospital Mortality, in a Cohort of Patients with COVID-19 in the Bronx, New York. Metabolism.

