# Supplementary material for: O Prolongamento do Intervalo QTc na Admissão está Associado ao Aumento da Mortalidade em Pacientes com SARS-COV-2 durante a Hospitalização
Source: Arq Bras Cardiol. 2022 Dec 20;120(1):e20220155. [Article in Portuguese] doi: 10.36660/abc.20220155 (PMC9833298; doi:10.36660/abc.20220155)
Supplement: Translation

# Translation: Prolongation of the QTc Interval at Admission is Associated with Increased Mortality in Patients with SARS-COV-2 during Hospitalization

**Authors:** Stephany Barbosa, Oscar Mauricio Muñoz, Alejandra Cañas, Angel Alberto Garcia

**Affiliations:** 1 Colombia Departamento de Medicina Interna - Pontificia Universidad Javeriana – Hospital Universitario San Ignacio, Bogotá – Colombia

**Keywords:** COVID-19, Mortality, Electrocardiography

## Abstract

**Background:**

Cardiovascular involvement associated with SARS-COV-2 infection is related to unfavorable outcomes during hospitalization. Therefore, the measurement at the admission of the QTc interval on the 12-lead electrocardiogram may be a prognostic marker.

**Objective:**

To identify the relationship between QTc prolongation at admission during hospitalization and mortality from SARS-COV-2.

**Method:**

Observational study based on a retrospective cohort of patients with confirmed SARS-COV-2 infection from San Ignacio University Hospital, Bogotá (Colombia), between March 19, 2020, and July 31, 2021. Mortality was compared in patients with prolonged and normal QTc at admission after controlling by clinical variables and comorbidities using bivariate and multivariate logistic regression models. A p-value <0.05 was considered statistically significant.

**Results:**

1296 patients were analyzed, and 127 (9.8%) had prolonged QTc. Mortality was higher in patients with prolonged QTc (39.4% vs 25.3%, p=0.001), as was hospital stay (median 11vs.8 days; p=0.002). In the multivariate analysis, mortality was associated with prolonged QTc (OR 1.61, 95% CI: 1.02; 2.54, p=0.038), age (OR 1.03, 95% CI 1.02; 1.05, p<0.001), male sex (OR 2.15, 95% CI 1.60; 2.90, p <0.001), kidney disease (OR 1.32, 95% CI 1.05; 1.66, p =0.018) and Charlson comorbidity index > 3 (OR 1.49, 95% CI 1.03; 2.17, p=0.035).

**Conclusions:**

Hospital mortality due to SARS-COV-2 is associated with prolonging the QTc interval at the time of admission, even after adjusting for age, sex, comorbidities, and basal severity of infection. Additional research is needed to establish whether these findings are related to cardiac involvement by the virus, hypoxia, and systemic inflammation.

## Introduction

Mortality in patients with SARS-COV-2 infection worldwide is close to 2%.^1^The number of cases continues to increase, with more than 250 million cases reported by November 2021.^2^Cardiovascular involvement associated with SARS-COV-2 infection is associated with a worse prognosis^3^and increased mortality. It is estimated that those with cardiovascular disease have a 3 times higher risk of suffering from the disease in severe form.^4^In particular, a high prevalence of cardiac arrhythmias in infected patients is described, suggesting that there is specific myocardial or conduction system involvement by the virus,^5^mediated mainly by inflammatory processes and hypoxia.^6^

The electrocardiogram is a useful tool to assess the degree of cardiac involvement given its wide availability, low cost, non-invasive nature and the possibility of immediate follow-up.^7^It has been described that up to 90% of hospitalized patients^8^present electrocardiographic alterations, including sinus tachycardia, QT prolongation, Torsades de Pointes, atrial fibrillation, nonspecific changes in ST and T wave, as well as conduction alterations or ventricular arrhythmias.

The corrected QT interval (QTc) prolongation depends on the variation in depolarization and ventricular repolarization.^9^However, the clinical relevance of these electrocardiographic findings associated with SARS-COV-2 infection is unknown, which beyond being related to arrhythmias, could become a useful risk marker.^10 , 11^

This study aims to identify the relationship between QTc prolongation at hospitalization and mortality from SARS-COV-2 during hospitalization, controlled by factors such as age, sex, and comorbidities, based on a cohort of patients treated in a highly complex hospital in Bogotá, Colombia.

## Method

An analytical observational study was conducted on a retrospective cohort based on the institutional registry of patients with SARS-COV-2 treated at the San Ignacio University Hospital (HUSI) in Bogotá, Colombia, between March 19, 2020, and July 31, 2021. We included all patients over 18 years of age hospitalized for SARS-COV-2 infection confirmed by PCR test, who had a 12-lead electrocardiogram taken at the entrance of hospitalization. The Ethics and Research Committee of the Pontificia Universidad Javeriana and the Hospital Universitario San Ignacio approved the study (Approval Code: FM-CIE-0883-21).

The information on sociodemographic variables, comorbidities, admission laboratories, and outcomes was systematically collected and stored in the confidential institutional registry using RedCap®. The rate of lost data was monitored to ensure data quality, and information from the extreme data was verified.

### Statistical analysis

For the analysis, the population was divided into two groups: patients with QTc interval within normal limits (QTc men < 470 msec and QTc women < 480 msec) and the second group with patients with prolonged QTc interval.

The Shapiro-Wilk test was used to evaluate the assumption of normality. The continuous variables did not present a normal distribution; therefore, we described the median and interquartile range (IQR). The comparison between the characteristics of the groups was performed using the Chi-square test for categorical variables or Mann-Whitney’s U test for continuous variables. A *p* -value less than 0.05 was considered statistically significant.

A logistic regression analysis was performed, taking mortality as the outcome. Initially, a bivariate analysis was performed and later, a multivariate analysis. The impact of the prolongation of baseline QTc was evaluated by controlling for the variables associated with mortality described in previous studies such as age, sex; comorbidities such as arterial hypertension, myocardial infarction, heart failure, diabetes mellitus, chronic kidney disease (CKD), chronic obstructive pulmonary disease (COPD), Charlson index (an assessment tool specifically designed to predict long-term mortality that has been used to demarcate prognostic differences between subgroups of patients who share the same medical diagnosis)^12^and use of drugs that prolong the QTc interval (hydroxychloroquine, chloroquine, azithromycin, lopinavir/ritonavir). A *p* -value less than 0.05 was considered statistically significant. The analysis was performed using a StataCorp statistical package. 2019. (Stata Statistical Software: Release 16. College Station, TX: StataCorp LLC).

## Results

We included 1296 patients with indication of hospitalization for SARS-COV-2, of which 127 (9.8%) had a prolonged QTc interval on the electrocardiogram taken at admission. Table 1 summarizes the demographic and clinical characteristics at the time of admission, comparing the groups according to the baseline QTc interval. The ages had no statistically significant differences. Males were more frequent among patients with prolonged QTc interval. Hospital stay was longer in the same group, with a median of 11 (IQR 6 - 19) vs. 8 days (IQR 4 - 16). The mortality during hospitalization was lower in patients with normal QTc (25.3% vs. 39.4%). An echocardiogram was performed during hospitalization in 241 patients (18.6%). Of these, the ejection fraction was greater than 50% in 58.6% and <40% in 22.6%, with similar characteristics in both groups.

**Table 1:** Clinical and demographic characteristics in the patients included according to the baseline QTc

Variable	QTc normal n = 1169	Prolonged QTc n = 127	p-value
Age, years, median (IQR)	63 (52–74)	67 (54–75)	0.065
Male, n (%)	637 (54.5)	81 (61.8)	0.045
**Comorbidities, n (%)**			
High blood pressure	480 (55.6)	67 (68.4)	0.016
Myocardial infarction	71 (8.2)	8 (8.2)	0.983
Heart failure	87 (10.1)	18 (18.4)	0.013
Diabetes Mellitus	208 (24.1)	24 (24.5)	0.932
Kidney disease	89 (10.3)	15 (15.3)	0.132
COPD	113 (13.1)	15 (15.3)	0.541
Charlson > 3, n (%)	362 (31.0)	41 (32.3)	0.761
**Admission laboratory, Median (IQR)**			
LDH, U/L	318 (237- 448)	414(288 -552)	<0.001
PCR, mg/dl	10.5(4.5-17.4)	10.5(7.3-18.7)	0.214
Dimer D, FEU/mL	871 (532-1553)	1233(659-2203)	0.006
Medications*	73 (6.2)	5 (4.0)	0.299
Days of hospitalization, median (IQR)	8 (4–16)	11 (6–19)	0.002
In-hospital death, n (%)	296 (25.3)	50 (39.4)	0.001

COPD: chronic obstructive pulmonary disease; IQR: interquartile range; U/L: units per liter; mg/dl: milligram per deciliter; FEU/mL: Fibrin equivalent units per milliliter. *At least 1 medication that prolongs QTc.LDH: lactate dehydrogenase; PCR: C-reactive protein.

Regarding the comorbidities of the included patients ( Table 1 ), arterial hypertension was the most frequent, followed by diabetes mellitus. The index of Charlson comorbidities was similar in both groups. However, a higher frequency of arterial hypertension and heart failure was found in the group of patients with prolonged QTc interval. The median values of LDH and D-dimer at admission were significantly higher among patients with QTc prolongation. There were no significant differences in the use of drugs that prolonged QTc.

The bivariate analysis ( Table 2 ) showed a significant increase in mortality associated with the prolonged QTc interval, age, and male sex. The comorbidities associated with increased mortality were: hypertension, kidney disease, COPD, and a Charlson index > 3.

**Table 2:** Bivariate and multivariate analysis of all included patients

Variable	Bivariate analysis		Multivariate analysis	

	OR (IC 95%)	p-value	OR (IC 95%)	p-value
QTc prolonged*	1.91 (1.31–2.80)	0.001	1.61 (1.02-2.54)	0.038
Age, years	1.05 (1.04-1.06)	< 0.001	1,03 (1,02-1,05)	< 0.001
Male**	1.87 (1.45-2.43)	< 0.001	2.15 (1.60-2.90)	< 0.001
**Comorbidities**				
High blood pressure	1.47 (1.12-1.95)	0.006	0.90 (0.65-1.25)	0.560
Myocardial infarction	1.01 (0.62-1.66)	0.946	0.63 (0.37-1.09)	0.102
Heart failure	1.19 (0.78-1.83)	0.402	0.70 (0.43-1.14)	0.157
Diabetes Mellitus	1.30 (0.95-1.77)	0.094	1.23 (0.89-1.72)	0.215
Kidney disease	1.45 (1.17-1.77)	< 0.001	1.32 (1.05-1.66)	0.018
COPD	1.81 (1.24-2.64)	0.002	1.13 (0.74-1.72)	0.559
Charlson >3	1.33 (1.27-1.40)	< 0.001	1.49 (1.03-2.17)	0.035
Medications***	1.59 (0.98-2.56)	0.060	1.04 (0.61-0.13)	0.871

COPD: chronic obstructive pulmonary disease; OR: Odds ratio; CI: confidence interval. * QTc > 480 milliseconds. ** Compared to women. *** At least 1 medication that prolongs QTc.

In the multivariate analysis ( Table 2 ), there was a significant association between mortality and prolongation of the QTc interval after control for age, male sex, kidney disease and Charlson comorbidity index > 3. The other included variables did not show statistical significance in the multivariate analysis.

## Discussion

Our study is the first conducted in Latin America and the largest reported so far, where we evaluate the relationship between in-hospital mortality and QTc prolongation at admission. We found a statistically significant difference in mortality in patients with prolonged QTc at admission after controlling for age, sex, and comorbidities.

Our results are similar to those reported by Farré et al., who analyzed 623 patients with SARS-COV-2, where 9.8% had prolonged QT, and found that death from any cause was higher in these patients (41.0% versus 8.7%, p < 0.001, HR 2.68, CI 1.58; 4.55).^10^Additionally, they support the results reported by Alsagaff in a meta-analysis that included seven studies, for a total of 2539 patients with SARS-COV-2 infection, who reported that a final outcome composed of ICU admission, severe disease and/or mortality more frequent among patients with QTc prolongation, (WMD 6.04; CI95%: 2.62; 9.45, p = 0.001; I^2^: 0%).^13^

It has been widely described that systemic inflammation and hypoxia in SARS-COV-2 infection can induce cardiac conduction abnormalities.^14 , 15^It has even been described as a simple marker that reflects the inflammatory state at the myocardial cellular level.^16^In our study, patients with QTc prolongation had additionally higher baseline values of inflammatory markers such as LDH and markers of endothelial damage with thrombotic involvement such as the D-dimer, which reinforces this hypothesis. Thakore et al. reported that patients with prolonged QTc intervals had a lower survival during hospitalization when compared to patients who had normal QTc.^17^This could be explained by immuno-mediated phenomena caused by the virus that result in a cytokine storm with elevated interleukin-6 (IL-6),^18 , 19^which blocks the potassium-related ether-a-go-go channel, which in turn increases circulating levels of IL-6, the above has been widely recognized in inflammatory diseases such as rheumatoid arthritis that are associated with QTc interval prolongation, and that improve with pharmacological management, for example, tocilizumab (anti-IL-6 monoclonal antibody).^20 , 21^

The use of drugs that prolong the QTc interval showed no statistically significant differences in hospital mortality, suggesting that QTc prolongation goes beyond a side effect of pharmacological origin.^16 , 22^Jiménez-Jáimez et al. analyzed 219 patients with electrocardiograms at admission. They described that outpatients who do not critically become ill with SARS-COV-2 treated with hydroxychloroquine, azithromycin, and antiretrovirals develop a non-relevant prolongation of the QT interval.^23^

In our study, we additionally found a relationship between QTc prolongation and a significant increase in hospitalization days, which could be related to greater severity, and associated complications. Other authors have suggested a finding, such as Thakore et al., who describe a longer duration of the QTc interval in hospitalized patients compared with discharged patients (450.1 ± 30.2 vs. 423.4 ± 21.7 msec, p < 0.0001).^17^

We found in the bivariate analysis that hospital mortality was associated with: older age, male sex, arterial hypertension, chronic kidney disease, COPD and Charlson index greater than 3, findings that were significant in the multivariate analysis for age, male sex, chronic kidney disease and Charlson index greater than 3, similar to that reported by Türkay et al., who included 419 patients in their study and divided them between surviving and non-surviving patients during hospitalization for SARS-COV-2 and described the paraclinical findings identified in the emergency room that could predict mortality, among these, patients with longer QTc intervals, generally did not survive.^24^

Our results suggest that the increase in mortality related to QTc prolongation was independent of the presence of comorbidities at admission. Also, Al-Zakhari et al. retrospectively analyzed 339 patients and demonstrated that the prolonged QTc interval has an independent and statistically significant relationship with mortality in patients hospitalized for SARS-COV-2 (r = -0.173, *p* < 0.05, 95% CI: -0.277, -0.064).^25^

Our study contributes to the growing medical knowledge related to the effects of SARS-COV-2 infection on the cardiac conduction system and the independent relationship of the prolonged QTc of the admission electrocardiogram as a prognostic marker of the disease, being an economical, available and easy to interpret in the emergency room.

As strengths of this study, we can mention a significant sample size, derived from the largest database by SARS-COV-2 reported so far in Colombia, with excellent quality and availability of information, as well as the absence of lost data. The main limitations describe biases related to the source of information, such as the self-reporting of comorbidities and the possibility of underestimating the real prevalence of comorbidities in this population. However, if there is a misclassification bias, this would be non-differential, which could dilute the size of the effect rather than increase it. Another limitation is that the registration of data to evaluate obesity, comorbidity strongly related to mortality,^26^was insufficient, so it was not included in the analysis. Likewise, information related to prolonging the QTc interval before infection or its behavior after it is unavailable. It is a limitation of our study that we could not identify whether the worse clinical outcome associated with prolonged QTc was related to ventricular dysfunction, ventricular arrhythmias, or cardiac structural damage at baseline, as we performed an echocardiogram just in a small fraction of patients. However, our main objective was to evaluate a factor associated with higher mortality, easily identifiable at the beginning of hospitalization, rather than to evaluate the mechanism of this association. Additional prospective studies would be necessary to clarify that point.

## Conclusions

Hospital mortality due to SARS-COV-2 is associated with prolonging the QTc interval at the time of admission, even after adjusting for age, sex, comorbidities, and basal severity of infection. Additional research is needed to establish whether these findings are related to cardiac involvement by the virus, hypoxia, and systemic inflammation.
